# Case of Fracture of the Skull

**Published:** 1884-07

**Authors:** Chas. A. Wall


### Case of Fracture of the Skull.

By Chas. A. Wall, M. S., M. D.

It is accepted that in punctured wounds of the skull the inner table is generally more broken and depressed than the outer, and in many cases the absolute necessity of surgical interference is only discovered in the course of the operation. Recognizing this fact, most writers upon surgery recommend the use of the trephine in these cases upon a different principle than in ordinary depressed fractures, and lay it down as a rule to apply the trephine in all cases of punctured wounds where there is any depression, whether there are signs of compression or not. A punctured wound being a compound one, the outer table is exposed, and from it the condition of the inner table can usually be judged, but at times cases will present themselves in which the advisability of an operation must be carefully considered, and the indications well weighed, before so serious a procedure as exposing the membranes of the brain is decided upon. Among this latter class I would report the following case: Chas. R., aged 45, German, a carpenter by trade, was a stout, healthy, thick-set man, of good family history, who was injured Nov. 2, 1882, by a brick falling upon his head from a distance of about thirty feet. He wore, at the time, a heavy, stiff felt hat, which may have somewhat deadened the force of the blow. He was rendered unconscious for a moment, but soon recovered and walked to my office, a distance of a quarter of a mile. Upon examination I found a triangular wound of the scalp one and one-half inches long, with the scalp loosened anteriorly an inch farther. At the anterior-superior angle the skull was depressed to the depth of three-sixteenths of an inch. The fracture was on the vertex and a little to the right of the median line, one-half of an inch square. This was not movable to any perceptible extent, but when subjected to slight pressure in making a digital examination, it caused him to vomit. Allowing him to become quiet I again made pressure with the same result. His pulse being full and slow and respiration impeded I determined to remove the depressed bone. Sending him to his home I operated, with the assistance of Drs. Stockton and Frederick, at 11 A. M., one and one-half hours after he received the injury.

An anæsthetic having been administered, the wound was enlarged an inch anteriorly and an endeavor made to remove the depressed fragment, but it was so held by the other portions overlapping that this could not be done. Including the depression in a large trephine, both tables were carefully removed, when a piece of bone from the inner table was found penetrating the membranes, upon withdrawing which free hemorrhage followed from the point of injury to such an extent that it seemed as though the superior longitudinal sinus had been opened. Careful examination, however, showed that the puncture was nearly an inch to the right of the median line. The inner table was rounded off and the wound cleaned. Hot water was applied to stop the bleeding, but as this did not control it, and it was venous in its nature, I concluded that the best thing to do under the circumstances would be to draw the scalp together and dress with a compress, watching for any symptoms due to pressure from a clot forming under it. Accordingly, the wound was drawn together by two sutures at the angles, leaving the inferior portion open for drainage. A compress was applied and the patient put to bed. One-sixth of a grain of morphia was given, to be repeated every three hours. When seen again, at 5 P. M., his pulse was 120, temperature normal and respiration regular. Upon removing the compress, there was no bleeding and the scalp had approximated through the whole extent of the wound.

November 3d, at 9.30 A. M., pulse 75, temperature 100°, and the wound appears to be uniting. From this time he could not be induced to maintain the recumbent position, as he said he felt as well as ever. A few days later I removed the stitches, as the scalp had entirely united by first intention. Within ten days he visited my office after walking five miles, and on the 15th of November he began to work at his trade. The spicula of bone which penetrated the membranes was pyramidal in shape, one-half an inch long, one-quarter of an inch wide and one-eighth of an inch thick, tapering to a sharp point.

It is remarkable that so serious an injury should heal so kindly, producing so little local or constitutional disturbance. When last seen, April 28, 1884, Mr. R. told me that he had been perfectly well and had suffered no inconvenience from the operation. The scalp was not tender to the touch, and was only slightly depressed where trephined. He had never found any ill effects from working in the sun in the hottest weather. He was in perfect health when injured and could not be impressed with the gravity of the operation. Feeling hungry during the night, he had arisen, gone into an adjoining room, and helped himself to some bread and butter. This probably accounts for the fact that his temperature went up to 100° on the morning of Nov. 3d, and it was the only time I found it above the normal. After the first day he was up and around the house, and it was almost impossible to keep him from going out, since, as he said, he soon felt as well as ever. In conclusion I would remark that the history of the case illustrates three facts:

1st. It may often be good surgery to trephine when the symptoms do not point to compression in so decided a manner as to warrant interference, for the condition of the inner table and membranes beneath can only be known after the removal of the outer table.

2d. Hemorrhage, even when alarming, if venous in character, may often be easily controlled. I stood ready, upon the first manifestation of pressure from a blood clot, to open the wound, and, if possible, place stitches in the torn vein, but happily it was not needed.

3d. The blood, filling up the place of the bone which was removed, did not cause any trouble, and was soon either absorbed or formed into organized tissue.

